# Management of Zinner's Syndrome Associated with Contralateral Seminal Vesicle Hypoplasia: A Case Report

**DOI:** 10.1155/2013/494215

**Published:** 2013-01-14

**Authors:** Mehdi Kardoust Parizi, Nasser Shakhssalim

**Affiliations:** Urology and Nephrology Research Center (UNRC), Shahid Labbafinejad Medical Center, Shahid Beheshti University of Medical Sciences (SBMU), No. 103, 9th Boustan, Pasdaran, Tehran 1666697751, Iran

## Abstract

A 27-year-old man presented with chronic hematospermia, painful ejaculation, and primary infertility. Physical examination, transrectal ultrasonography, and pelvic magnetic resonance imaging (MRI) demonstrated left seminal vesicle cyst, left renal agenesia, and contralateral seminal vesicle hypoplasia. Hormone workup (LH, FSH, prolactin, and testosterone) was normal. Sperm analysis showed oligoasthenozoospermia and low ejaculate volume. We performed transurethral resection of the ejaculatory duct (TUR-ED) using methylene blue vasography guidance without surgical-related complications. Hematospermia and painful ejaculation completely improved at 2-month followup, and the patient's wife experienced a missed abortion thereafter. This patient was considered as a rare variant of Zinner's syndrome and was managed effectively with a less invasive treatment modality (TUR-ED).

## 1. Introduction

Zinner's syndrome is a congenital malformation of the seminal vesicle and ipsilateral upper urinary tract that includes seminal vesicle cyst, ejaculatory duct obstruction, and ipsilateral renal agenesia [1]. In this paper, we present an unusual variant of this rare condition and describe management of the patient's chief complaint (refractory hematospermia).

## 2. Case Report 

A 27-year-old man presented with hematospermia and painful ejaculation since 8 months ago. He also suffered from irritative lower urinary tract symptoms and primary infertility. Digital rectal examination (DRE) revealed a large bulging originating from near the prostate. The patient had normal bilateral testes and vas deferens on physical examination. 

Semen analysis showed ejaculate volume of 1 mL and oligoasthenozoospermia (total sperm count of 10,250,000 and nonmotile sperm of 99%). Hormone analysis, including serum prolactin, luteinizing hormone (LH), follicle-stimulating hormone (FSH), and testosterone, was within the normal limits.

**Figure 1:**
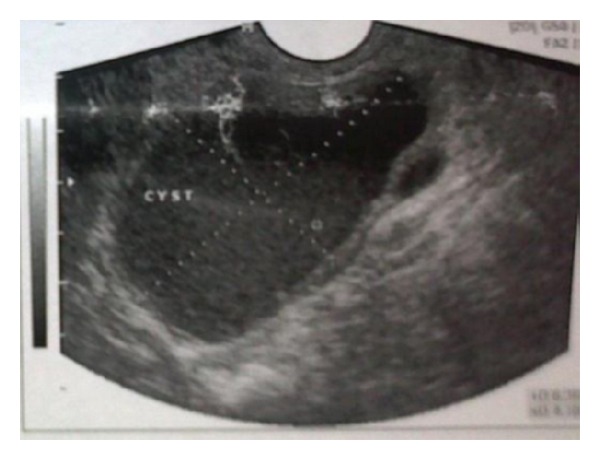
Transrectal ultrasonography of the left seminal vesicle cyst.

**Figure 2:**
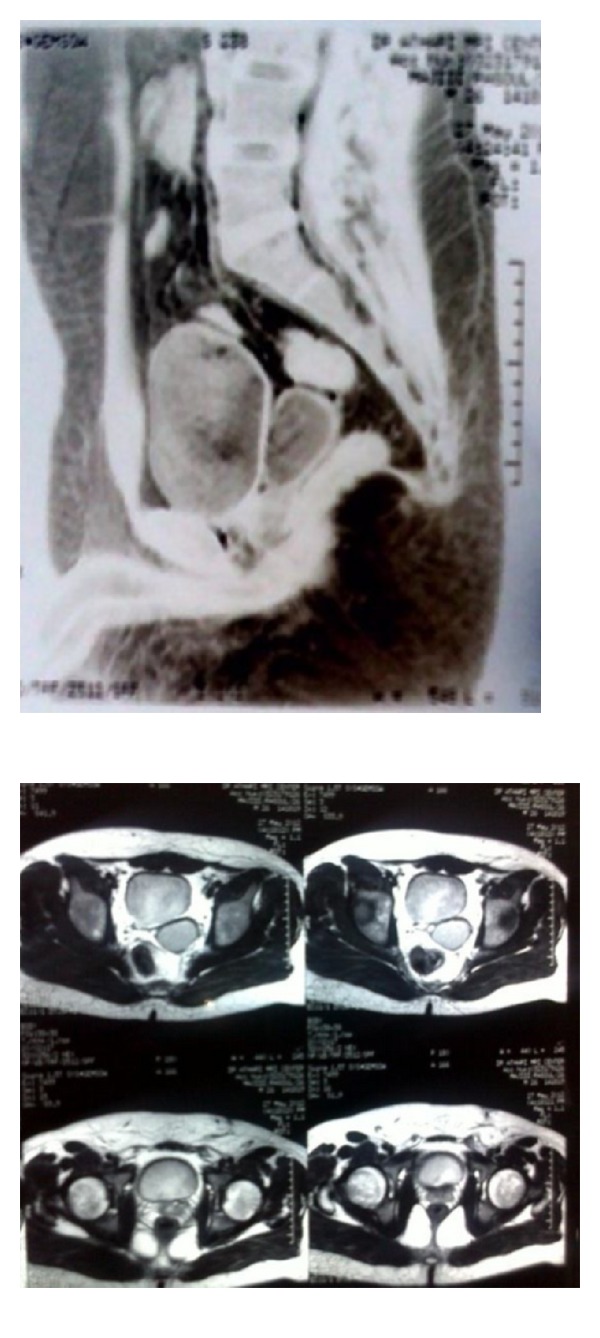
Pelvic MRI showing a large cystic structure in the left seminal vesicle and right seminal vesicle hypoplasia.

Transabdominal ultrasonography showed left kidney agenesis and contralateral compensatory hypertrophy. Transrectal ultrasonography demonstrated a large cystic mass in the left seminal vesicle area, but the right seminal vesicle was not visible (Figure 1). Pelvic MRI was performed without contrast media, which revealed a large cystic structure containing hemoglobin degradation products in the left seminal vesicle measuring 5 × 3.5 cm. Right seminal vesicle was hypoplastic. The prostate and bladder appearance was normal (Figure 2).

Diagnostic cystoscopy revealed normal appearance and position of the right ureteral orifice, but the left ureteral orifice was not found. Bulging effect of the left seminal vesicle cyst was noted on the prostatic urethra near the verumontanum during urethroscopy.

On the basis of these findings, we reported a very rare variant of Zinner's syndrome that was associated with contralateral seminal vesicle abnormality (right seminal vesicle hypoplasia). This case underwent medical therapy, including antibiotic, *α*-blocker, and anti-inflammatory agents, for several weeks, but no satisfactory response was observed. Therefore, we preferred surgical treatment. Among different treatment modalities, including cyst puncture, transurethral cyst resection, laparoscopic, and open surgeries, we recommended transurethral resection of the ejaculatory duct (TUR-ED) as a definitive therapy.

We delivered the left vas deferens through an incision over the left hemiscrotum. Then, diluted methylene blue (20 mL) was injected into the vas deferens through a fine angiocatheter to evaluate correct position of the left ejaculatory duct orifice in the prostatic urethra during simultaneous rigid urethroscopy. In the next step, the seminal vesicle cyst wall was resected using a cutting loop through a rigid 24 Fr cystourethroscope.

The patient was discharged the day after the procedure. After 2-month followup, hematospermia and painful ejaculation completely improved. Although his wife experienced a missed abortion after this treatment, no increase in ejaculation volume has occurred. 

## 3. Discussion

Since 1914 that Zinner reported the association between seminal vesicle cyst and ipsilateral renal agenesia [2], several researchers have published their experience with diagnosis and management of this rare syndrome [1–6]. Zinner's syndrome is characterized by triad of the ejaculatory duct obstruction, seminal vesicle cyst, and ipsilateral renal agenesia. Fewer than 100 cases with this diagnosis were reported in literature [7, 8].

 The seminal vesicle is originated from the mesonephric duct under the effect of testosterone [9]. It seems that abnormality occurs before the 7 weeks of gestation, when the ureteric bud appears; hence, it affects the ureteric bud formation and results in maldevelopment of wolffian duct structure, such as seminal vesicle [10]. The case presented in this study is unique because the abnormality was seen in contralateral genital structures (combination of left seminal vesicle cyst and right seminal vesicle hypoplasia).

Some investigators recommend vasovesiculography as the diagnostic test of choice. They perform cyst aspiration and contrast injection; however, this is rarely done unless the patient is symptomatic [5]. In a study, Roehrborn et al. concluded that pelvic ultrasonography may be cost-effective and also accurate in most patients [6]. In our study, we performed vasography using methylene blue to confirm effective resection of the obstructed ejaculated duct orifice. 

Of less invasive diagnosis modalities, several methods can be used to evaluate this rare condition. MRI may be assumed as ideal imaging study to evaluate malformations of the mesonephric duct due to its multiplanar ability, appropriate soft tissue resolution, and use of nonionizing radiation [3, 11, 12]. In our study, MRI enabled us to exactly evaluate the lower genitourinary system and confirm seminal vesicle cyst and contralateral seminal vesicle hypoplasia.

Patients suffering from Zinner's syndrome are usually symptomatic in the 2nd to 4th decade of their life [5]. Genitourinary symptoms, including irritative and obstructive lower urinary tract symptoms, perineal pain, painful ejaculation, and hematospermia, are common chief complaints. 

Several optional treatments are available for this rare anomaly. Existence of bothersome symptoms is a significant factor that can affect the decision for treatment. Most investigators recommend treatment only for symptomatic patients [3, 4]. We have faced different treatment modalities, including medical treatment, percutaneous drainage, transurethral aspiration and alcohol injection, transrectal aspiration, laparoscopy, and even open cyst surgery in the literature [3–5, 7, 13, 14]. Kajita et al. reported effectiveness of percutaneous seminal vesicle cyst drainage in a patient with Zinner's syndrome. They found no recurrence at 5-month followup [13]. Van den Ouden et al. reviewed diagnosis and management of 52 patients with Zinner's syndrome in a pooled analysis. They reported cure rate of 100% and 75% for open surgery and transurethral unroofing of the cyst, respectively. They also concluded that cyst aspiration should be used only for diagnosis due to its low success rate (30%) [5]. Kao et al. performed transrectal aspiration of the seminal vesicle cyst in a patient with bladder outlet obstruction due to seminal vesicle cyst. They noted improvement in the lower urinary tract symptoms and increase in mean urinary flow rate after the procedure [14]. Seo et al. performed laparoscopic surgery with transperitoneal approaches as a minimally invasive modality in the management of 4 patients with congenital seminal vesicle cyst associated with ipsilateral renal agenesis. They noted mean hospital stay of 6.8 days and no operative complications or transfusions [7].

We think that cyst formation in the seminal vesicle may be as a result of distal obstruction; hence, removal of this factor may result in clinical improvement. Therefore, TUR-ED can be assumed as a safe and effective treatment in the management of symptomatic and also medical treatment refractory seminal vesicle cyst. 

## 4. Conclusion

Ipsilateral seminal vesicle cyst and renal agenesia and contralateral seminal vesicle hypoplasia rare variants of Zinner's syndrome, and symptomatic cases can be managed safely and effectively using TUR-ED.
